# Psychometric evaluation of the Chinese version of fear of hospitalization scale among outpatients: A validation study

**DOI:** 10.3389/fpsyg.2022.1095905

**Published:** 2023-01-11

**Authors:** Wenbo Li, Hongyu Yu, Yanli Zhang, Bing Li, Mingshu Fu

**Affiliations:** ^1^Department of Nursing, Jinzhou Medical University, Jinzhou, China; ^2^Department of Dermatology, Shengjing Hospital of China Medical University, Shenyang, China; ^3^Department of Neurosurgery, The First Affiliated Hospital of China Medical University, Shenyang, China

**Keywords:** fear, hospitalization, outpatients, psychometric validation, cross-cultural adaptation, factor analysis

## Abstract

**Background:**

In China, some patients avoid seeking medical care and are highly sensitive to subsequent medical care because of fear of possible hospitalization after a diagnosis has been established. Early identification of fear of hospitalization is essential for clinical staff to develop targeted education and interventions. However, there are currently no tools to assess outpatients’ fear of hospitalization in mainland China. This study aimed to translate the Fear of Hospitalization (FH) scale into Chinese and verify its reliability and validity in outpatients.

**Methods:**

Through convenience sampling, 664 outpatients who required hospitalization were recruited from two cities in Liaoning Province, China. The reliability of the translated scale was measured by internal consistency, split-half reliability, and test–retest reliability. The validity of the translated scale was evaluated by expert consultation, exploratory factor analysis, and confirmatory factor analysis. Data were analyzed using SPSS 25.0 (IBM Corp., Armonk, NY, United States) and AMOS 23.0 (IBM Corp., Armonk, NY, United States).

**Results:**

The Cronbach’s α value of the Chinese version of the FH scale was 0.849, and the Cronbach’s α value of the dimensions ranged from 0.857 to 0.902. The test–retest reliability value of 0.868 shows good temporal stability. The split-half reliability value of 0.910 indicates a high degree of measuring the same content. The content validity index of the scale (S-CVI) was 0.924, indicating a good level of content validity. The 3-factor structure supported by eigenvalues, total variance explained, and scree plot was obtained using exploratory factor analysis. In addition, all recommended fit indicators were within the acceptable range by confirmatory factor analysis.

**Conclusion:**

The Chinese version of the FH scale is valid and reliable in outpatients. The developed three-factor structured scale will help identify outpatients with a high fear of hospitalization and can inform the development of educational intervention plans for care managers, physicians, and nurses. In addition, it helps clinicians and nurses take action to reduce this fear of hospitalization in patients and prevent avoidance of using health care services due to fear of hospitalization.

## Introduction

Hospitalization is a stressful event for patients of every age and can lead to a vicious cycle of patients delaying treatment and seeking alternative therapies out of fear of hospitalization, and only agreeing to be hospitalized for treatment when their disease escalates (Lowey et al., 2014; Raju and Reddy, 2017; Zvonareva et al., 2021; Correale et al., 2022). Fear of medical treatment is defined as the emotional and psychological response to a medical event experienced by a person in a hospital environment and is a common stress reaction during outpatient clinic visits (Steward and Steward, 1981; Lyu et al., 2020). Studies have shown that patients’ perceptions of pain, threat, and unknown prognostic risks associated with medical events can lead to medical fear, often accompanied by motor agitation and somatic autonomic dysfunction, resulting in anxiety, fear, panic, panic attacks, excessive sweating, and trembling of the hands and feet (Cornelius et al., 2018; Chang, 2019; Hadasik et al., 2021). During hospitalization, patients may feel a lack of privacy and severe limitations on self-direction, loss of personal space, and freedom to move around because they can no longer do anything for themselves and others (Weltens et al., 2021; Huang et al., 2022). They also feel helpless, angry, and insecure because of separation from their loved ones (Hall et al., 2022; Tavakoli et al., 2022). Outpatients are well aware that hospitalization means they will undergo previously unanticipated investigations, procedures, and treatments, some of which may be invasive, painful, or invasive of personal dignity, or even pose a threat to safety, creating a fear of hospitalization and possibly leading to delayed admission or rejection of hospital care. Therefore, assessing patients’ fear of hospitalization in a clinical setting is interesting and necessary.

Patient fear is a normal and complex emotional response, often caused by patient speculation about whether surgery will be successful and how well they will recover from hospitalization, and is essentially a fear of physical harm and death (Ji et al., 2022). The fear of surgery often presents as an emotional state before surgery, and it is recognized as one of surgery’s most significant psychological and socioeconomic risks (Celik and Edipoglu, 2018; Wu et al., 2019). Surgical fear has adversely affected patients’ psychosocial and physical recovery, such as increased pain after surgery, poor physical function, or mental health issues (Wilson et al., 2016; Theunissen et al., 2018). In addition to the surgery, people fear undergoing anesthesia, blood transfusions, being pricked by needles, and even losing their dignity (Kilinc and Ozer, 2017; Mohan et al., 2017). In a study investigating fear of anesthesia, it was shown that the primary sources of fear were fear of postoperative pain (84%), fear of not being able to wake up after surgery (64.8%), fear of nausea or vomiting (60.2%), and fear of drains and needles (59.5%). Patients were less concerned about being paralyzed by anesthesia (33.5%) or exposing personal problems (18.8%). In addition, the study also showed that gender influenced fear of anesthesia, with women being more fearful than men (Mavridou et al., 2013). In addition to patient concerns about surgery and anesthesia, patients experience fear due to a lack of communication and contact with hospital staff and information about various tests, medications, and therapeutic care operations (Tiwary et al., 2019). Conflicting instructions and plans from different healthcare providers can also cause patients to feel confused, fearful, and uncertain of correct statements (Nikitara et al., 2020). The closed management of wards during the prevention and control of the new coronary pneumonia epidemic is an effective means of preventing infection, cutting off transmission routes, and ensuring the health of hospitalized patients. Still, separation from friends and family may negatively impact hospitalized patients, leading to a significantly increased incidence of anxiety and depression (Ma et al., 2020). Thus, the concept of fear of hospitalization can be further applied to multicomponent fears, consisting of the following components: fear of injury (physical or mental) inevitably associated with appropriate diagnostics or treatment, fear of injury caused by diagnostic or treatment errors, fear of being humiliated as a person due to loss of privacy and autonomy in making decisions, and fear to be isolated from the persons with whom they have strong emotional ties. Fear of harm and medical procedures may prevent patients from seeking medical care when they are ill (Cavallo and Forman, 2020; Tesfaw et al., 2020; Kim et al., 2022). It is a severe public health problem that patients often avoid hospitalization despite physician recommendations (Pan et al., 2016).

Currently, there is only one scale to assess medical fear in children in China: the CMFS (Children’s Medical Fear Scale; Broome et al., 1988). Therefore, in many studies, non-specific questionnaires have been used to assess patients’ psychiatric problems related to hospitalization, such as the State–Trait Anxiety Inventory (STAI), Beck Anxiety Inventory (BAI), Hamilton Anxiety Rating Scale (HAM-A), Zung Self-Rating Anxiety Scale (SAS), Depression Anxiety Stress Scale (DASS), and Surgical Fear Questionnaire (SFQ; Hamilton, 1959; Zung, 1971; Beck et al., 1988; Spielberger, 1989; Lovibond and Lovibond, 1995; Theunissen et al., 2014). However, existing instruments for assessing fear of hospitalization are either limited in their target population or too general, and there is currently no scale that comprehensively assesses fear of hospitalization in outpatients in China. Based on the above, there is a need for reliable instruments to assess patients’ fear of hospitalization in China objectively. Professor Slobodan M. Jankovic developed and validated the FH scale (Jankovic et al., 2018) based on the guidelines developed by DeVellis (1991). Therefore, this study aimed to translate the FH scale into Chinese and validate its psychometric properties. This study also provides an instrument to quickly and accurately measure the fear of hospitalization among Chinese outpatients.

## Materials and methods

### Study design

This study was a cross-sectional and observational survey designed to test the reliability and validity of the new Chinese version of the Fear of Hospitalization scale, also known as the FH scale.

### Participants

Convenience sampling was conducted from March to July 2022 in the two Chinese cities of Shenyang and Jinzhou to recruit eligible outpatients. Inclusion criteria were outpatients who were literate, over 18 years of age, and had obtained informed consent. Exclusion criteria were outpatients who were pregnant, lactating, cognitively impaired, emotionally disturbed, mentally retarded, and had incomplete patient records. Ultimately, 664 outpatients were recruited from the hospital through convenience sampling with the assistance of the Director of Nursing based on available conditions. We collected basic information about the socio-demographic characteristics of the participants (including age, sex, education level, place of residence, living conditions, previous experience with surgery in general anesthesia, and clinical diagnosis).

### Translation, back-translation, and transcultural adaptation of the FH scale

Our translation work has obtained Professor Slobodan M. Jankovic’s permission. The Brislin double back-translation method was adopted to translate the FH scale (Brislin, 1970). First, two Chinese professors majoring in English translated the FH scale into Chinese. Then, two foreign teachers who were native English speakers did the reverse translation. In addition, psychological experts were invited to make cultural adjustments to the translated scale to make the items more compatible with Chinese expressions habits. The draft of the Chinese version of the FH scale was finally formed.

Some modifications have been made to the items in the scale to fit the Chinese cultural context, known as cultural adaptation. (1) Expert consultation: two psychiatrists, two psychologists, one clinical care manager, one English language specialist, and one nurse researcher with experience in acculturation and validation studies for quality life instruments, all with graduate degrees and above. The final version was generated after adapting and modifying each item in the first draft of the Chinese version according to Chinese cultural and linguistic habits. (2) Pretest: A convenience sampling method was used to select 10 outpatients for a preliminary survey, and each participant was invited to evaluate the layout design and understanding of each item. It is important to note that the researchers explained the purpose and significance of the study to respondents before sending the scales and obtaining their informed consent. Outpatients reported that items on the scale were easy to understand, and the scale structure was clear. Finally, the Chinese version of the FH scale was completed.

### Questionnaire design

#### Background characteristics

Our general demographic characteristics questionnaire was developed following a systematic literature review and rigorous team negotiation. Seven variables were included in the questionnaire: age, gender, education level, place of residence, living conditions, previous experience with surgery in general anesthesia, and clinical diagnosis.

#### The FH scale

The FH scale is a 17-item scale developed by Dr. Slobodan M. Jankovic et al. to comprehensively measure the fear of hospitalization experienced among outpatients (Jankovic et al., 2018). The FH scale includes three dimensions: Factor 1: Fear of being injured (7 items), Factor 2: Mistrust to the medical staff (5 items), and Factor 3: Fear of losing privacy, autonomy, and family ties (5 items). In addition to positive wording, there is also negative wording (items 2, 9, 10, and 11 are reverse scored, while the rest are forward scored). Based on a five-point Likert scale, each item is rated (5 = disagree; 4 = partially disagree; 3 = Neither agree nor disagree; 2 = partially agree; 1 = completely agree). The total and dimension scores are the sum of each item’s scores, with the total score ranging from 17 to 85. A higher score indicates a weaker degree of fear of hospitalization experienced by outpatients. According to the investigators (Cronbach’s alpha 0.799), there is acceptable internal consistency in the original scale and the patients themselves (Cronbach’s alpha 0.760).

#### The surgical fear questionnaire

Several questionnaires like Hospital Anxiety and Depression Scale (HADS), Hamilton depression scale (HAMD), Hamilton Anxiety Scale (HAMA), Self-rating Anxiety Scales (SAS), Self-rating Depression Scale (SDS), Generalized Anxiety Disorder 7-item Scale (GAD-7), Patient Health Questionnaire 9-item Scale (PHQ-9), and many others have been developed to evaluate specific components of mental problems among patients related to hospitalization. Most outpatients refuse to be hospitalized for treatment because of their fear of surgery, and surgical procedures can cause anxiety and fear in patients who believe they will threaten their physical changes and psychological and social reactions to hospitalization (Garcia et al., 2018). Due to its close relationship to hospitalization fear, the SFQ was used in this study to assess criterion validity, which was developed to evaluate the level of fear of surgical intervention. In 2019, Wu Jun translated and localized the Chinese version of the scale, which was verified to have good reliability and validity (Wu and Zhu, 2019). This scale is a self-rating scale consisting of 2 dimensions with eight items. A Likert 10-point scoring system (not at all afraid to very afraid) was used to collect responses from outpatients. The score ranges from 8 to 80. Generally, the higher the score, the stronger fear of undergoing surgery among patients. The Cronbach’s α value was 0.968, with domains ranging from 0.56 to 0.82.

### Data collection

With the assistance of the director of nursing, the researcher explained the study’s purpose and significance and recruited participants. The nursing director arranged a quiet classroom for participants to complete the translated scales anonymously. Seven hundred ten outpatients were recruited to participate in the survey, and 679 outpatients agreed. It was finally decided to retain 664 questionnaires after removing those with missing data from the sample. We asked 50 outpatients to complete the translated scale again after 2 weeks to assess the test’s reliability.

### Statistical analysis

SPSS 25.0 (IBM Corp., Armonk, NY, United States) and AMOS 23.0 (IBM Corp., Armonk, NY, United States) was used to complete the statistical analysis. Frequency and composition ratios were used to describe the general demographic characteristics of outpatients. We evaluated the items’ quality using item analysis and the content validity of the scales using expert consultation. Exploratory factor analysis (EFA) was performed using the principal axis factoring method to explore the underlying factor structure of the translation scales. A confirmatory factor analysis was conducted using AMOS 23.0 to examine the structural validity of the scale. Internal consistency analysis and test–retest reliability analysis were conducted to determine the scale’s homogeneity and stability.

### Item analysis

We used the critical ratio, correlation coefficient, and Cronbach’s α if item deletion method to evaluate each item’s suitability. A *t*-test was performed between the high group (highest 27%) and the low group (lowest 27%; McCowan and McCowan, 1999) to determine whether the item discriminated between the high and low groups. The absolute *t*-values were generally removed if they were below 3 (Raykov and Marcoulides, 2016). In addition, we calculated item-total correlation coefficients to determine item homogeneity, and item-total correlation coefficients ≥ 0.4 are considered appropriate for item homogeneity (Raykov and Marcoulides, 2016). The Cronbach’s α coefficient was calculated after removing each item to determine the items’ quality. It is suggested that you delete an item if its Cronbach’s coefficient increases significantly after its deletion, meaning the item decreases internal correlation and should be removed (Raykov and Marcoulides, 2016). It was done to determine if the items of the translated scale could be retained.

### Reliability analysis

Our test–retest reliability and internal consistency analyses were conducted on the translated Chinese FH scale. For the internal consistency analysis, Cronbach’s coefficient and split-half reliability coefficients were calculated for the translated scale and its dimensions to evaluate the homogeneity of the items. The split-half reliability of the translated scale was assessed by dividing the items into two parts in odd and even order, and the correlation between the results of both sides of the scale was calculated (Ren et al., 2021). After 2 weeks, 50 previously diagnosed outpatients were remeasured with the translated scale, and correlation coefficients were calculated to evaluate how stable the scale had become. All three Cronbach’s coefficients, the split-half reliability coefficient, and the test–retest reliability coefficient had to be at least 0.7 to satisfy our requirements (Chang et al., 2018).

### Validity analysis

A panel of experts consisting of three psychiatrists, two psychologists, one surgeon, and one internist assessed the content validity of the translated scales. These experts were selected based on: (i) their extensive expertise in psychiatry, psychology, and clinical medicine; (ii) their familiarity with the manual steps of the scale and psychometric measures; (iii) their bachelor’s degree or higher and least 10 years of experience in the field; (iv) their rigorous and pragmatic approach to research; and (v) their volunteers to participate in this study. The experts’ answers were collected using a four-point Likert scale (1 = not relevant, 2 = weakly relevant, 3 = strongly relevant, 4 = highly relevant). Not relevant and weakly relevant are assigned 0 points, and strongly relevant and highly relevant are assigned 1 point. An item’s content validity index (I-CVI) is determined by the percentage of experts who scored three or four out of the total number of participants. S-CVI is the content validity index of the scale, which is the average of the I-CVI of each item in the scale. There has been an indication of good content validity when the I-CVI ≥ 0.78 and the S-CVI ≥ 0.90, indicating that the overall content validity of the scale is good (Shi et al., 2012).

The underlying factor structure of the translation scale was evaluated using exploratory factor analysis (EFA) and confirmatory factor analysis (CFA). We randomly divided the sample of 664 cases into two groups, one group (*n* = 332) for the EFA and the other group (*n* = 332) for the CFA. In general, the characteristics of the two groups were similar. Principal axis factorization of EFA was completed, and the dataset was considered suitable for EFA only if the Bartlett test for sphericity was significant (*p* < 0.05; Bartlett, 1954) and the KMO > 0.60 (Kaiser H. F., 1970; Kaiser H. F. J. P., 1974) to explore the underlying factor structure of the scale. In addition, principal component analysis (PCA) with varimax rotation was used in EFA to estimate the principal components of the analysis. It was recommended that each item should have a factor loading of 0.40 or higher and no cross-loadings (Alavi et al., 2020; Schreiber, 2021). The corresponding items would be deleted if these requirements were not met (Gorsuch, 1997). Generally, contributions over 50% are considered acceptable, and contributions over 70% are deemed suitable (Diamond et al., 2014). The model fitting index for the translation scale was examined using an Analysis of Moment Structure (AMOS). The following metrics must be good to confirm the replicability of the first-order three-factor structure of the FH scale: χ^2^/DF, the goodness-of-fit index (GFI), the adjusted goodness-of-fit index (AGFI), the incremental fit index (IFI), the Tucker Lewis index (TLI), and the comparative fit index (CFI). Typically, χ^2^/DF is required to be <3, while all other values are required to be >0.9, indicating good fit of the model (Bentler and Bonett, 1980; Gorsuch, 1997). Furthermore, the root means a square error of approximation (RMSEA) should be <0.08, indicating a good fit and a good model fit (Schreiber et al., 2006). Additionally, convergent and discriminant validity tests were performed to determine the scale’s structural validity. We used the average variance extracted (AVE) values and the composite reliability (CR) values to measure convergent validity. An acceptable model should have a CR > 0.7 and an AVE > 0.45 (Kline, 2015). For assessing discriminant validity, we calculated the square root of the AVE value and the correlation coefficient for each factor. Our requirement was that the square root of the AVE value exceed the correlation coefficient between the corresponding factors (Fornell and Larcker, 1981).

### Convergent validity

**Figure 1:**
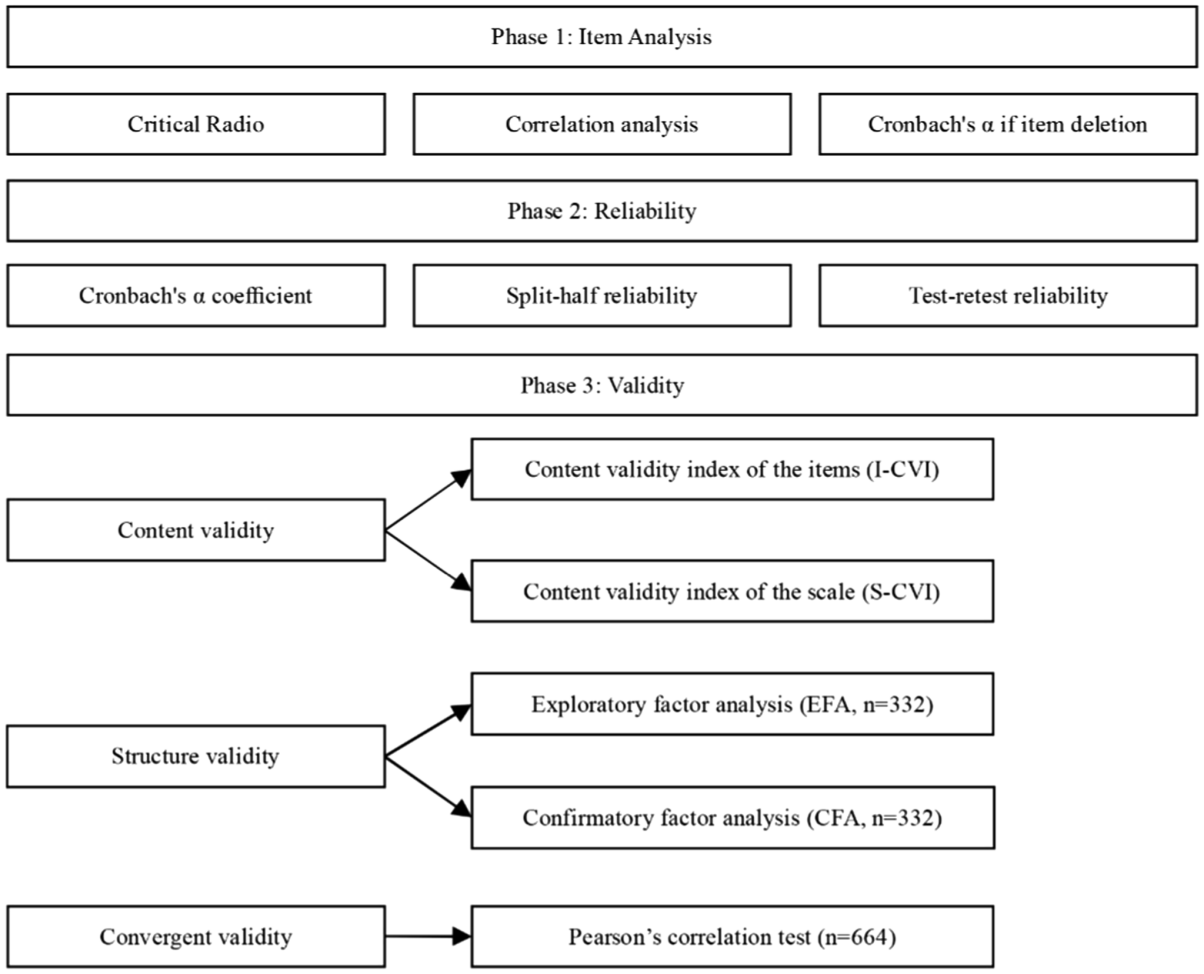
The data analysis procedure for Chinese version of the FH scale.

The calibration correlation is calculated to measure the degree of correlation between the measuring and standard scales using a recognized valid scale as a standard. This study used the SFQ as its calibration standard. A Pearson correlation analysis was conducted to determine whether the Chinese version of the FH scale was correlated with the SFQ. The test has high validity when the correlation coefficient r > 0.70. The validity of the test is moderate when 0.4 < r < 0.7. When r < 0.4, the validity of the test is low (Wang and Sun, 2011). Figure 1 illustrates the steps for the statistical analysis of the data.

### Ethical considerations

The Jinzhou Medical University Ethics Committee approved the study protocol (Ref: JZMULL2022025). The authors of the original study authorized the use of the FH scale. Before taking part in the survey, participants received an explanation of the purpose and significance of the study and were asked to sign an informed consent form. The questionnaires returned were all anonymous. The survey was conducted confidentially to ensure the confidentiality of the data.

## Results

### The general demographic characteristics

This study included 664 outpatients: 283 men (42.6%) and 381 women (57.4%). Participants aged > 35 years accounted for 49.8%. More than half (78.9%) of the participants lived in urban areas; 39% had primary education or below; the largest proportion of the participants lived with their families (89.9%). The following Table 1 provides additional sociodemographic information.

**Table 1:** Frequency distribution of demographic characteristics (*n* = 664).

Factors	Group	*n*	%
Age	<25	120	18.1
	25–35	213	32.1
	>35	331	49.8
Sex	Men	283	42.6
	Women	381	57.4
Education level	Primary education and below	259	39.0
	Junior secondary education	153	23.0
	High school education/Technical secondary school education	53	8.0
	Technical secondary school education	140	21.1
	Undergraduate education and above	59	8.9
Place of residence	Urban	524	78.9
	Rural	140	21.1
Living conditions	Living alone	67	10.1
	Living with family	597	89.9
Previous experience with surgery in general anesthesia	Yes	374	56.3
	No	290	43.7
Clinical diagnosis	Hypertension	135	20.3
	Chronic heart failure	48	7.2
	Coronary disease	18	2.7
	COPD	33	5.0
	Asthma	137	20.6
	Diabetes mellitus	21	3.2
	Cancer	28	4.2
	Surgical disease (any disease that requires surgical treatment)	180	27.1
	Other	13	2.0
	No diagnosis of a chronic disease	51	7.7

### The translation and revision of the FH scale

The original Chinese version of the FH scale was developed through translation, back translation, and cultural adaptation. Seven experts were invited to conduct an expert consultation on the draft Chinese version of the FH scale. The scale included three dimensions (Fear of being injured, Mistrust of the medical staff, Fear of losing privacy, autonomy, and family ties) and 17 items. According to all 10 outpatients in the initial survey, the revised draft of the Chinese version of the FH scale was easy to understand and answer. Eventually, 17 items were developed as a pretest version of the Chinese version of the FH scale.

### The exploration and analysis of the item

The item’s quality was estimated based on the critical ratio, the item-scale correlation coefficient, and Cronbach’s coefficient. A critical ratio (CR) > 3.000 indicates a high discriminability of items. The critical ratios of items in the translation scale range from 11.459 to 20.375 (*p* < 0.001), indicating good discriminability of each item. This study’s item-total correlation coefficients ranged from 0.446 to 0.588 (*p* < 0.001). Taking the translated scale and removing each item, we found that Cronbach’s coefficient for the translated scale was 0.835–0.847, not more than Cronbach’s coefficient for the scale itself (0.849). Therefore, each item can be accepted without deletion.

### The psychometric evaluation of the FH scale

#### Content validity

Seven qualified experts were invited to assess the content validity of the translation scales (I-CVI and S-CVI), and the results showed a range of 0.857 to 1.000 for I-CVI and 0.924 for S-CVI on the translation scale. The results suggested an adequate content validity of the questionnaire.

#### Construct validity

**Figure 2:**
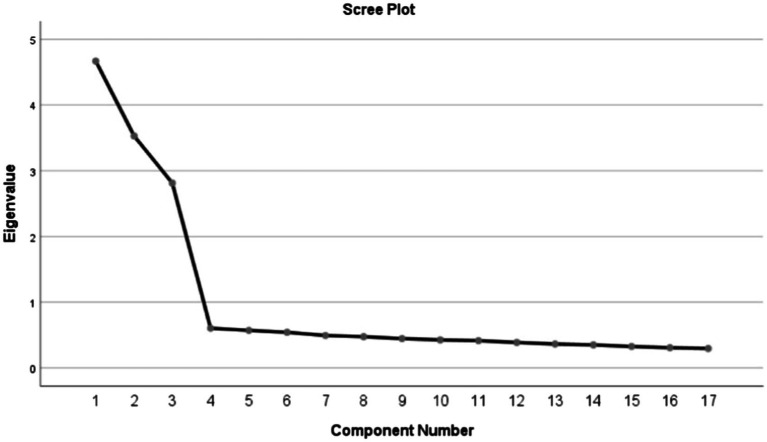
Screen plot of exploratory factor analysis for the Chinese version of the FH scale.

In the EFA, the Kaiser–Meyer–Olkin value was 0.887, and the Bartlett sphericity test was significant (χ^2^ = 2725.797; *p* < 0.001). Due to this, the matrix is not an identity matrix, and factor extraction can be performed with it. Kaiser’s rule showed that the three-factor model explained 64.788% of the total variance with initial eigenvalues > 1 each. A scree plot further confirmed the three-factor structure within the original scale, showing that the descending tendency weakened after the third point (Figure 2). Based on the results of the varimax rotation, three factors were identified which could explain 27.474%, 20.764%, and 16.550% of the variance, respectively. Furthermore, the factor loadings of the factors are also satisfactory and are displayed in Table 2.

**Table 2:** Factor loadings of exploratory factor analysis for the Chinese version of the FH scale.

Item	Factor 1	Factor 2	Factor 3	Communality scores
a1	**0.806**	−0.012	0.026	0.650
a2	**0.766**	0.001	0.084	0.594
a3	**0.789**	0.009	0.054	0.625
a4	**0.761**	−0.038	0.045	0.582
a5	**0.801**	−0.021	0.049	0.645
a6	**0.754**	0.077	0.113	0.587
a7	**0.773**	0.047	0.082	0.606
a8	0.076	0.052	**0.813**	0.669
a9	0.058	0.031	**0.818**	0.674
a10	0.109	0.115	**0.774**	0.624
a11	0.102	0.044	**0.832**	0.704
a12	0.030	−0.007	**0.789**	0.624
a13	−0.041	**0.835**	0.075	0.704
a14	0.027	**0.831**	−0.026	0.692
a15	0.079	**0.812**	0.050	0.668
a16	0.014	**0.823**	0.062	0.681
a17	−0.033	**0.823**	0.073	0.685
Eigenvalues	4.671	3.530	2.814	
Percentage of variance	27.474	20.764	16.550	

Major loadings for each item are bolded.

**Figure 3:**
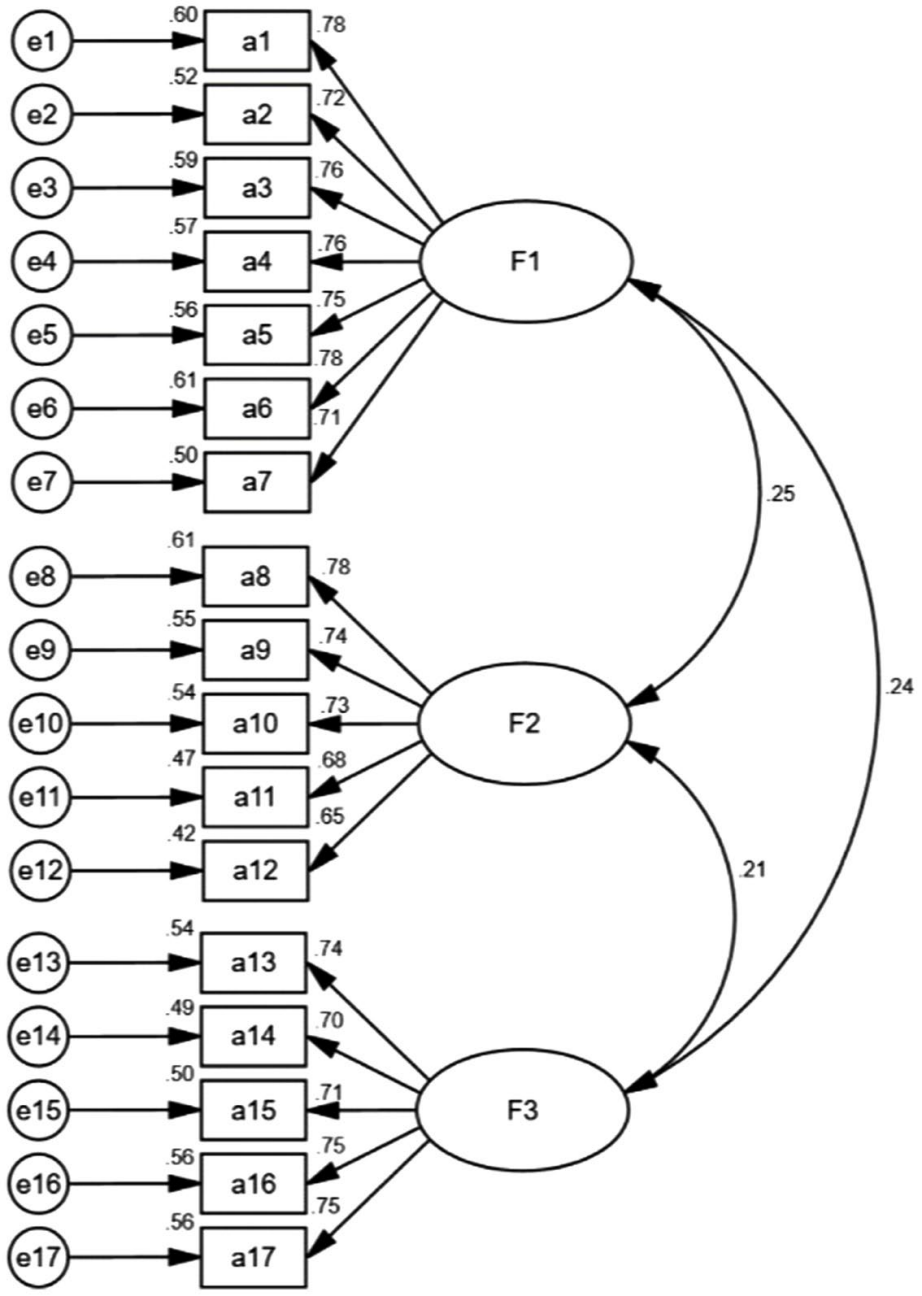
Standardized three-factor model of the Chinese version of the FH scale.

According to the CFA analysis, the three-factor model was fitted using the maximum likelihood SEM (Figure 3). In terms of the CFA results, χ^2^/DF = 1.764, GFI = 0.932, AGFI = 0.910, RMSEA = 0.048, TLI = 0.958, CFI = 0.964, IFI = 0.965, PGFI = 0.707, and PNFI = 0.787. There is no doubt that the three-factor model fits appropriately based on the fit indices selected. According to the convergent validity analysis, the AVE values ranged from 0.516 to 0.564, and the CR values ranged from 0.841 to 0.900. A discriminant validity analysis revealed that the square root values of AVE ranged from 0.718 to 0.751, greater than the correlation coefficients between the corresponding factors in Table 3.

**Table 3:** Convergent validity and discriminant validity of the FH scale.

Factors	Correlation between factors			**AVE**	**Sqrt (AVE)**	**CR**
	Factor 1	Factor 2	Factor 3			
Factor 1	1			0.564	0.751	0.900
Factor 2	0.254	1		0.531	0.718	0.850
Factor 3	0.239	0.210	1	0.516	0.729	0.841

#### Criterion validity

We used the Chinese version of the SFQ as the criterion scale to measure the FH scale’s criterion validity. As a result of the correlation analysis, the total scores of the two scales were negatively correlated (r = −0.649, *p* < 0.001). The significant correlation coefficients for the different dimensions and the SFQ were −0.481, −0.416, and −0.401, respectively (*p* < 0.001).

### Internal consistency, split-half reliability, and test–retest reliability

An analysis of the stability and homogeneity of the Chinese version of the FH scale was conducted based on internal consistency, split-half reliability, and test–retest reliability. The translated scale had a Cronbach’s alpha value of 0.849, while Cronbach’s α values of the dimensions ranged from 0.857 to 0.902. The split-half reliability of the scale was 0.910. A random sample of 50 outpatients was selected for retesting after 2 weeks, and the test–retest reliability was 0.868 (Table 4).

**Table 4:** Reliability analysis for the Chinese version of the FH scale.

The scale and its dimension	Score	Cronbach’s alpha	Split-half reliability	Test–retest reliability
The FH	54.22 ± 7.65	0.849	0.910	0.868
Fear of being injured	22.60 ± 4.81	0.902		
Mistrust to medical staff	16.05 ± 3.28	0.857		
Fear of losing privacy, autonomy, and family ties	15.57 ± 3.19	0.869		

## Discussion

The FH scale was adapted cross-culturally to measure outpatients’ fear of hospitalization, and its reliability and validity were tested on 664 outpatients in this study. The FH scale was first applied to a Chinese population and has good construct validity, discriminant validity, and reliability. It can be used to predict the fear of hospitalization and to identify and screen patients diagnosed by physicians or who need to be hospitalized for treatment purposes but avoid hospitalization because of fear. It also facilitates the development of health education interventions by healthcare professionals, essential to reduce or eliminate patients’ fear of hospitalization and increase their acceptance of hospitalization.

This study’s FH scale was translated into Chinese and cross-culturally adapted according to the Brislin translation principle (Brislin, 1970). Seven experts adjusted the first translation based on their professional knowledge, clinical experience, and language expression habits, and eventually, the Chinese version of the FH scale was developed. The Chinese and original scales were fully demonstrated to be equivalent in terms of equivalence.

There has been an extensive review of the content validity of the items by seven experts, and the experts have agreed that the scale, in its original configuration, showed good content validity, with all experts agreeing on the scale items. In the preliminary survey, 50 outpatients indicated that the structure of the Chinese version of the FH scale was simple and reasonable, with clear semantic expressions and easy-to-understand content. The final Chinese version of the FH scale was divided into three dimensions and contained 17 items. The CR values in the item analysis were much higher than the standard values. In addition, the items of the questionnaire were well distinguished, and the scores of each item had a moderate to high correlation with the scale’s total score (Huang et al., 2020). In addition, after deleting each item, Cronbach’s α value did not exceed the original values of the translated scale. In conclusion, the content and structure of the Chinese version of the FH scale are reasonable, 17 items can be retained, and no further items need to be added or reduced.

As part of the reliability analysis conducted in this study, the internal consistency reliability, split-half reliability, and test–retest for reliability analysis were measured to assess the consistency and stability of the Chinese version of the scale. Cronbach’s α coefficient can reflect the homogeneity between all items in the scale (Tavşancıl, 2002; Şencan, 2005). The results showed that the Cronbach’s α coefficient of the Chinese version of the scale was 0.849, and the Cronbach’s α coefficient of each dimension was 0.857 to 0.902, slightly higher than the results of the original scale (Celik and Edipoglu, 2018), indicating that the Chinese version of the scale had a higher internal consistency of the items. Similarly, it was found that the split-half reliability coefficient was 0.910, confirming the previous conclusion. The test–retest reliability is the consistency of results produced by repeating measurements on the same group of subjects using the same research instrument, which can represent the test’s stability and consistency over time (Leppink and Pérez-Fuster, 2017). According to the results, the test–retest reliability of the Chinese version of the scale was 0.868, indicating that the Chinese version of the FH scale has good stability and can be used to measure the fear of hospitalization behavior in outpatients. Overall, the Chinese version of the FH scale has shown good reliability among outpatients.

The validity of the Chinese version of the FH scale was evaluated based on its content validity, construct validity, and calibration validity. Seven experts evaluated the Chinese version of the questionnaire for its content validity. The results showed that the I-CVI was 0.857 to 1.000 and the S-CVI was 0.924, better than the standard values of 0.78 and 0.9, respectively (Lenz, 2010). Thus, the scale showed good content validity. Construct validity, also known as conceptual validity, reflects the extent to which a scale is integrated with the theoretical or conceptual framework on which it is based. The evaluation of outcome validity is mainly based on factor analysis, which can be divided into exploratory factor analysis (EFA) and confirmatory factor analysis (CFA). When evaluating the structural validity of a questionnaire using EFA, the structural validity of the questionnaire is satisfactory if the cumulative variance contribution of the common factors extracted from the scale is >60% and the loadings of the items on their imputation factors are >0.4 (Carlsson et al., 2016; van Bruggen et al., 2018). In this study, the EFA results showed that the three-factor structure extracted from the Chinese version could explain the total variation well, explaining 64.788% of the total variance, the factor loadings of all items in the questionnaire were >0.4, and the factor attributions of every item were consistent with the original version (Celik and Edipoglu, 2018). The results suggested that the Chinese version was conceptually adequate and had appropriate construct validity. It indicates that the items have a strong explanatory power for assessing the fear of hospitalization among outpatients. Meanwhile, the fit index of the Chinese version of the scale was within the acceptable range and stronger than the original version in CFA (Celik and Edipoglu, 2018), indicating that the Chinese version of the FH scale has a good overall fit. In terms of calibration validity, the high correlation between the FH scale and the SFQ (r = −0.649, *p* < 0.001) also indicated that the scale had appropriate calibration validity. In conclusion, we believe that the Chinese version of the FH scale has appropriate validity among outpatients.

## Limitation and perspectives

In this study, we considered several limitations that need attention and discussion. First, despite the study’s sample size meeting the criteria, a large multi-center sample should be considered for improved sample representation and to investigate cultural differences across regions. Second, this study relied on self-reported questionnaires, and bias is inevitable. Finally, although we have adequately validated the psychometric characteristics of the Chinese version of the FH scale in outpatients, we have not yet explored the factors that influence fear of hospitalization behavior. Therefore, this will serve as the focus of our future work, which will be very important for our next steps.

## Conclusion

After translation and cultural adaptation, the FH scale was successfully introduced into China, and its psychometric properties have been validated. In response to the serious public health problem of patients avoiding hospitalization despite physician advice, the developed questionnaire can provide a reference for care managers, physicians, and nurses to identify patients at high risk for fear of hospitalization and to develop educational programs and interventions to improve behaviors to prevent fear of hospitalization among outpatients, as well as a basis and prerequisite for research related to fear of hospitalization among outpatients.

## Data availability statement

The original contributions presented in the study are included in the article/supplementary material, further inquiries can be directed to the corresponding author.

## Ethics statement

The studies involving human participants were reviewed and approved by the Jinzhou Medical University Ethics Committee (Ref: JZMULL2022025). The patients/participants provided their written informed consent to participate in this study.

## Author contributions

WL, HY, YZ, BL, and MF were involved in the study route design and data collection. After data collection and analysis, WL wrote the draft. HY and BL made essential revisions to the draft to identify important intellectual content. YZ and MF performed data collection and statistical analysis. All authors contributed to the article and approved the submitted version.

## Conflict of interest

The authors declare that the research was conducted in the absence of any commercial or financial relationships that could be construed as a potential conflict of interest.

## Publisher’s note

All claims expressed in this article are solely those of the authors and do not necessarily represent those of their affiliated organizations, or those of the publisher, the editors and the reviewers. Any product that may be evaluated in this article, or claim that may be made by its manufacturer, is not guaranteed or endorsed by the publisher.
